# EmbryoMiner: A new framework for interactive knowledge discovery in large-scale cell tracking data of developing embryos

**DOI:** 10.1371/journal.pcbi.1006128

**Published:** 2018-04-19

**Authors:** Benjamin Schott, Manuel Traub, Cornelia Schlagenhauf, Masanari Takamiya, Thomas Antritter, Andreas Bartschat, Katharina Löffler, Denis Blessing, Jens C. Otte, Andrei Y. Kobitski, G. Ulrich Nienhaus, Uwe Strähle, Ralf Mikut, Johannes Stegmaier

**Affiliations:** 1 Institute for Automation and Applied Informatics, Karlsruhe Institute of Technology, Karlsruhe, Germany; 2 Institute of Toxicology and Genetics, Karlsruhe Institute of Technology, Karlsruhe, Germany; 3 Institute of Applied Physics, Karlsruhe Institute of Technology, Karlsruhe, Germany; 4 Institute of Nanotechnology, Karlsruhe Institute of Technology, Karlsruhe, Germany; 5 Department of Physics, University of Illinois at Urbana-Champaign, Urbana, Illinois, United States of America; 6 Institute of Imaging and Computer Vision, RWTH Aachen University, Aachen, Germany; Hebrew University of Jerusalem, ISRAEL

## Abstract

State-of-the-art light-sheet and confocal microscopes allow recording of entire embryos in 3D and over time (3D+t) for many hours. Fluorescently labeled structures can be segmented and tracked automatically in these terabyte-scale 3D+t images, resulting in thousands of cell migration trajectories that provide detailed insights to large-scale tissue reorganization at the cellular level. Here we present EmbryoMiner, a new interactive open-source framework suitable for in-depth analyses and comparisons of entire embryos, including an extensive set of trajectory features. Starting at the whole-embryo level, the framework can be used to iteratively focus on a region of interest within the embryo, to investigate and test specific trajectory-based hypotheses and to extract quantitative features from the isolated trajectories. Thus, the new framework provides a valuable new way to quantitatively compare corresponding anatomical regions in different embryos that were manually selected based on biological prior knowledge. As a proof of concept, we analyzed 3D+t light-sheet microscopy images of zebrafish embryos, showcasing potential user applications that can be performed using the new framework.

This is a *PLOS Computational Biology* Software paper.

## Introduction

Development of animals is the result of an intricately orchestrated interplay of cell division, migration, differentiation and death [1]. Mapping the origin and the fate of cells and their descendants is essential to understand the developmental mechanisms underlying the self-construction of complex metazoan body plans. Especially, a detailed qualitative and quantitative analysis of cell behavior (*e.g.*, migration, division) in space and time is key to understand the steps in cell specification and fate determination that precede overt cellular differentiation and organ formation [2–6].

To capture these dynamic developmental processes at the single-cell level in entire complex biological systems, light-sheet-based microscopy platforms have been extensively used [7–11] and are being further evolved to image fast, clearly and deep with improved illumination and detection methods [12–14]. Imaging transgenic organisms that selectively express one or more fluorescent reporters in cell nuclei or plasma membranes allow studying tissue morphogenesis and cell dynamics in unprecedented detail [3, 13, 15–18]. These types of recordings, however, generate an enormous amount of imaging data over time, which can easily accumulate data sets of 10 terabytes and more, depending on the spatial and temporal resolution and the duration of recording. Thus, handling and analysis of terabytes of time-resolved 3D (3D+t) microscopy images and the building of representative virtual models of embryos for finding and tracking biologically relevant groups of cells is a major technical issue [4, 6, 11].

Existing approaches to cope with such large-scale data sets essentially comprise methods for automatic detection, segmentation and tracking of fluorescently labeled cell nuclei or plasma membranes. These tools enable quantitative reconstructions of cell shape changes [15, 19, 20] and to create digital cell lineages of entire embryos [4, 8, 21–28]. Furthermore, software tools like TeraFly, BigDataViewer or ParaView offer nice visualizations of large-scale image data sets but the possibilities to quantitatively analyze cell movement trajectories are limited [29–31]. Most of these workflows are available as open-source software and can be used to reconstruct cell lineages, to visualize the results and to quantitatively analyze morphogenesis in the early embryo [4, 9, 21, 28, 32–35]. Although these workflows were already successfully applied to different biological organisms, such as fruit fly, zebrafish or mouse embryos [4, 19, 33], the analyses were mostly constrained to test particular hypotheses including predefined region of interest selections and, due to a lack of interactivity, on-the-fly analyses were impossible. Moreover, despite the recent breakthroughs in 3D+t microscopy of biological organisms, state-of-the-art segmentation and tracking algorithms still struggle with producing error-free lineages in the presence of high cell density, requiring manual corrections of identified track fragments [4, 33, 35, 36]. Hitherto, resulting cell lineages were thus mostly analyzed by using a few manually curated selections and offline visualizations, which is time consuming and is not suitable for experiments comprising multiple biological repeats. An interactive and systematic approach that allows identifying groups of interest based on available prior knowledge and that can be easily transferred to other data sets is still missing.

Based on preliminary ideas in our previous work [37, 38], we developed the new software tool EmbryoMiner to make huge 3D+t tracking data sets accessible in a user-friendly and intuitive way, which we believe provides a solution to numerous manual lineage analyses conducted in the past [39–56]. EmbryoMiner can be used to navigate and focus within the wealth of cell trajectories, to derive new hypotheses on the fly, to interactively group the data on the foundation of existing prior knowledge and to apply data mining methods such as clustering and classification for automatic group identification. Following the idea of linking and brushing [57], the possibility to select and analyze detected cell trajectories at arbitrary time points or based on trajectory features among multiple synchronized visual representations makes new types of experiments possible. For instance, the new framework enables interactive retrospective cell fate mapping or a virtual dissection of an entire organism that are impossible using conventional fate mapping and dissection approaches. A set of interactive editing tools is provided that can be used for efficient data curation of erroneous tracks and we provide data importers to integrate with the existing segmentation and tracking solutions. As a proof of principle, we provide application examples for all developed components and demonstrate the capabilities of our framework by interactively separating and analyzing hypoblast and epiblast cells during zebrafish gastrulation in four different wild-type embryos and by semi-automatically repairing tracks of precursor cells of the olfactory epithelium of a zebrafish embryo. We anticipate that our framework will significantly increase the possibilities of interactively analyzing huge amounts of trajectory data in an efficient new way and will help gathering new quantitative insights in embryonic development easily and fast.

## Design and implementation

We developed EmbryoMiner, a new interactive analysis framework featuring responsive 3D visualizations, semi-automatic selection strategies, data curation possibilities and a powerful data analysis back end in order to interactively visualize, annotate and analyze huge amounts of cell tracking data in a user-friendly and intuitive way as described in the following sections. To demonstrate the capabilities of EmbryoMiner, we prepared two data sets that were acquired using 3D+t light-sheet microscopy, one for analyzing cell movement trajectories at the whole organism level and another one for focusing on neural crest cell development, a subset of the cell population (see S1 Note for a brief overview of the sample data sets and S2 Note for detailed materials and methods).

### Ethics statement

Zebrafish (*Danio rerio*) wild-type embryos expressing a fluorescent marker in the cell nucleus *Tg(h2afva:h2afva-GFP) kca66Tg* and neural crest reporter line *Tg(-7.2sox10:h2afva-Eos)* were used in this study. Zebrafish husbandry and experimental procedures were performed in accordance with German animal protection regulations (Regierungspräsidium Karlsruhe, Germany, AZ35-9185.81/G-137/10).

### Implementation details

EmbryoMiner was implemented on the foundation of the open-source data mining toolbox SciXMiner for MATLAB [58, 59]. For improved user-friendliness, all methods are accessible through a graphical user interface that allows analyzing data in an efficient way. Due to the limited interactivity of MATLAB visualizations when dealing with large-scale 3D+t data sets, we developed a new visualization framework based on the Visualization Toolkit (VTK, http://www.vtk.org/). The framework is based on a new bidirectional interface between MATLAB and VTK using local TCP sockets and custom callbacks that allows interactive 3D+t trajectory data exploration directly from SciXMiner. Application-specific data visualizations can be easily created to handle user interaction and to process inputs. SciXMiner provides full analytical power to explore the data and the VTK interface is optimally suited to visualize huge amount of complex 3D data. The bidirectional interface allows one to create and control 2D and 3D visualizations in an interactive way. To get a modular and easily expandable software interface for the visualization tasks, the VTK dependencies are separated in a C++ interface. This interface is completely independent from MATLAB, allowing to reuse the algorithms also for possible other interfaces or in other programming languages. All visualized objects are accessible using unique IDs, in order to access and change properties of the objects and to create arbitrary selections of groups of interest. The generic design of the visualization framework permits integrating new visualization windows that are automatically connected to all other existing windows with custom-tailored data representations. To integrate with existing tracking approaches, we implemented importers for tracking data obtained with TGMM [4], BioEmergences [33], TrackMate [60] and any algorithm that produces results in the Cell Tracking Challenge format [24, 36] (S1 Video).

### Visual analysis of spatiotemporal cell migration patterns

Identifying spatiotemporal patterns in large-scale trajectory databases is an almost impossible challenge without a convenient tool at hand that allows one to focus on a particular region or phenomenon of interest. As a cornerstone of our newly developed framework, we thus implemented a set of highly interactive trajectory data visualizations that enable an effective interaction with huge 3D+t data sets. The different types of visualization allow focusing on various aspects of the data and the most suitable or multiple complementary data representations can be selected to optimally support the respective analysis task.

**Fig 1:**
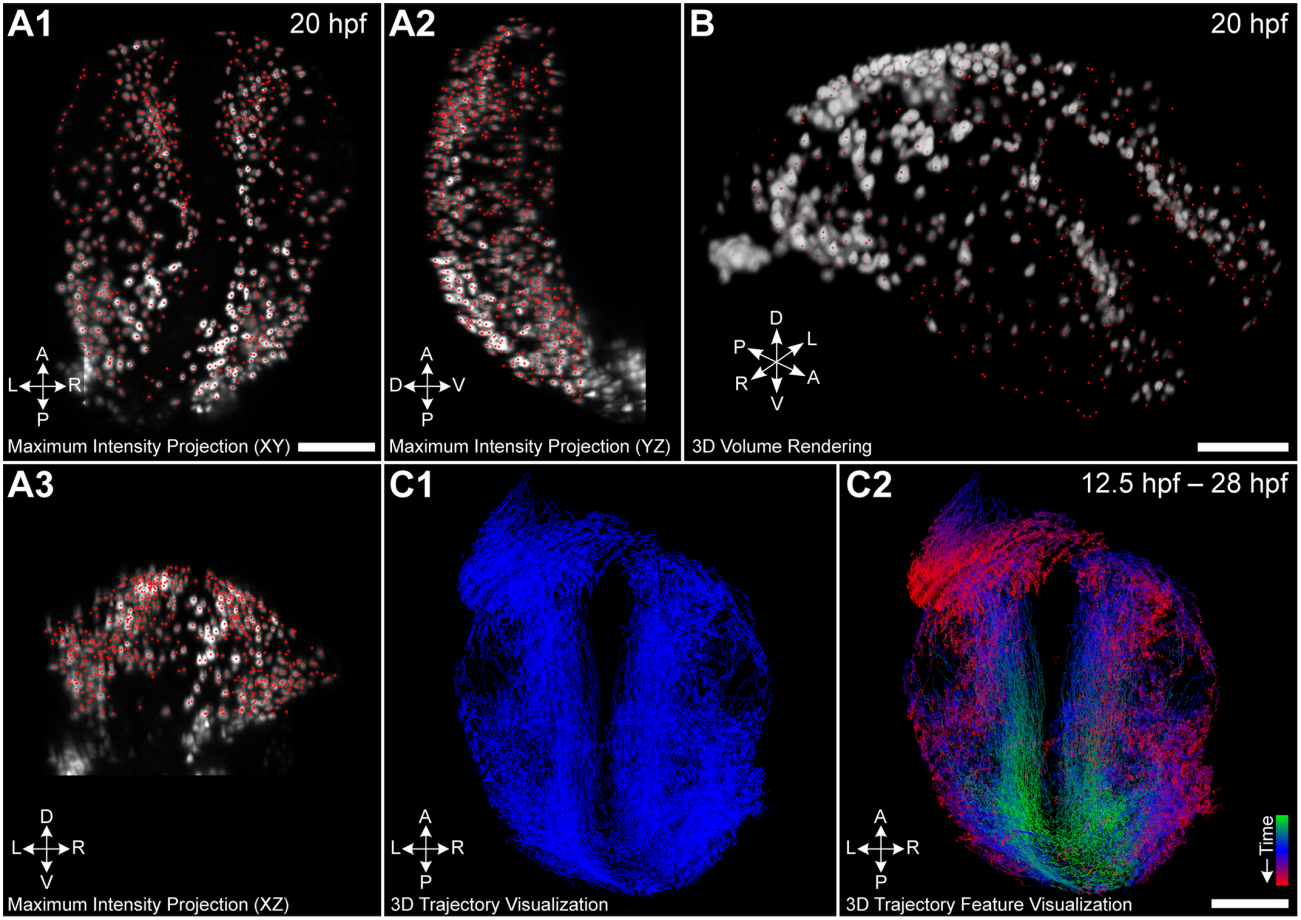
Different possibilities to visualize 3D+t cell tracking data. (A) Temporally scrollable maximum intensity projection with superimposed centroids of detected cell nuclei (red dots) allows following development over time. (B) Similar to the maximum intensity projections, detected nuclei can be superimposed on interactive 3D volume rendering of the original data. (C) Tracks of all cells can be analyzed from arbitrary orientations in an interactive and highly responsive 3D visualization using plain colors (C1) or quantitative trajectory features (C2, color-code indicates time). The panels show neural crest cells of a zebrafish embryo at 20 hpf (A, B) and the trajectories spanning the entire experimental duration from 12.5 − 28 hpf (C). Scale bar: 100 *μ*m.

We implemented (1) a maximum intensity projection overlay of the raw images and the tracking results, (2) the tracks of the moving cells in 3D, (3) a window containing only the selected tracks in 3D for a more detailed analysis of an isolated group of cells and (4) a GPU-accelerated volume rendering module that allows to view the tracking data directly in the spatiotemporal 3D+t context of the raw images. In Fig 1 and S2 Video, we show maximum intensity projections (Fig 1A) and 3D volume renderings (Fig 1B) of the neural crest cells of a zebrafish embryo (20 hpf) and the corresponding 3D+t cell migration trajectories (Fig 1C). In the maximum projection view and the 3D volume rendering view, detected cell centroids at each time point or the tracks are superimposed on a 2D projection of the raw images along the x-, y- or z-axis, or directly rendered on the original 3D microscopy data. In both visualization modes, the user can easily scroll through all time points of the data set and zoom to a specific region of interest to visually analyze cell migration patterns from a global scale down to the single-cell level. The possibility of visualizing cell tracks in 3D additionally provides insights into the global movement of the cells to investigate the movement paths spanning the entire experimental duration. The user can interact with the data from different perspectives and points in time to get familiar with the characteristics of the data, to derive new hypotheses on the fly or to interpret morphological changes in the embryo. Moreover, selected trajectories can be visualized in a separate 3D window to focus on a subset of the trajectories.

### Quantitative description of cell movement behavior

In addition to the qualitative visual analysis of the trajectory data, a major advantage of the digital representation of cell tracks is the possibility to quantitatively characterize the movement behavior of individual cells or groups of cells. We provide a set of single features (one scalar value or vector per track) that comprises global measures including length, center of gravity of all spatial locations, average movement angle change or average speed, to name just a few. Furthermore, time series features (one separate scalar value for each time point of a track) may be used to analyze temporally changing properties of a track, such as speed, density, directional changes, distance to a reference point and the like. An overview of trajectory features that we re-implemented in the SciXMiner-based extension package is provided in [25, 35]. Once a project has been loaded to SciXMiner, all implemented features can be added to the project via the graphical user interface and will be computed instantly.

**Fig 2:**
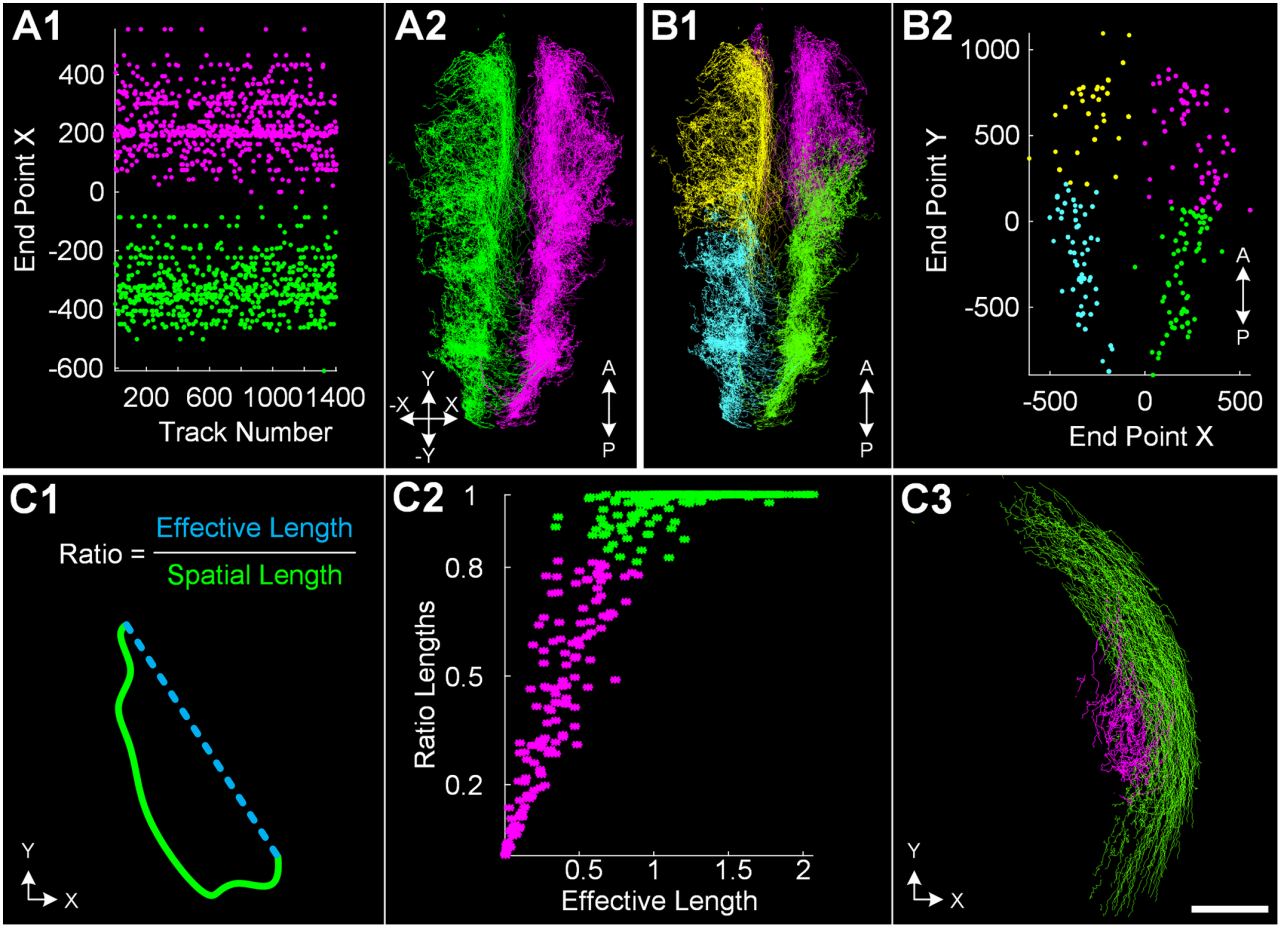
Feature-based extraction of groups of interest. (A) Quantitative features associated with each of the cell tracks allows feature-based selections. The left and right part of an embryo was separated along the anteroposterior axis using the end point coordinates of each track. (A1) and (A2) show the identified groups of ∼1400 neural crest cells as a scatter plot (x coordinate of the trajectory end points versus the unique track ID) and a 3D rendering of all trajectories. (B) Cluster algorithms can be used to automatically group the data. The example shows four identified clusters using the end point locations in the XY-plane at a selected time point as a 3D rendering (B1) and a scatter plot (B2). (C) Special trajectory features can be used to characterize particular cell movements in the early embryo. (C1) schematically illustrates the ratio of effective displacement (distance between start and end point) versus the spatial length (integrated path length) that was used to automatically identify two clusters corresponding to hypoblast cells (magenta) and epiblast cells (green) visualized as scatter plot (C2) and 3D rendering (C3). Panels (A) and (B) show neural crest cells of a zebrafish embryo (12.5 − 28 hpf) and panel (C) is based on a slice cut from a whole-embryo zebrafish data set (5 − 7.25 hpf). Scale bar: 100 *μ*m.

Calculated single features and time series can be used for colorization of cell trajectory data in one of the visualization windows to visually analyze quantitative properties on top of the original data (S3 Video). Moreover, we implemented a 2D scatter plot window to visualize selected trajectory features and to apply feature-based data selection strategies. As a simple example, Fig 2A and 2B shows how neural crest cells of a zebrafish embryo may be split into different spatial regions using the end points of tracks in the investigated time interval and Fig 2C shows the feature-based separation of hypoblast and epiblast cells in a cross-section of a whole-embryo data set.

Feature-based selections can either be based on manually specified thresholds or by using the MATLAB-based SciXMiner back end of the framework to apply data mining methods such as clustering, interactive filtering and classification. Fig 2B shows the result of searching four clusters in the neural crest data set based on the spatial x- and y-coordinates of the cells at a single time point. Fig 2C shows a custom-tailored cell trajectory feature, where we separate hypoblast (magenta) and epiblast (green) cells in a cross-section of the whole-embryo data set based on the ratio of effective displacement (distance between start and end point) *vs.* the integrated path length (sum of the velocity vector magnitudes). Two groups of cells were identified using a clustering algorithm and the final grouping is indicated by the color code. To cope with potentially imperfect single-cell measurements (*e.g.*, caused by discretization artifacts or inhomogeneous marker expression), the SciXMiner back end allows to combine single-cell measurements of selected groups of cells to more robust measures such as the median, quantiles or mean time series.

### Virtual dissection of large-scale cell tracking data

**Fig 3:**
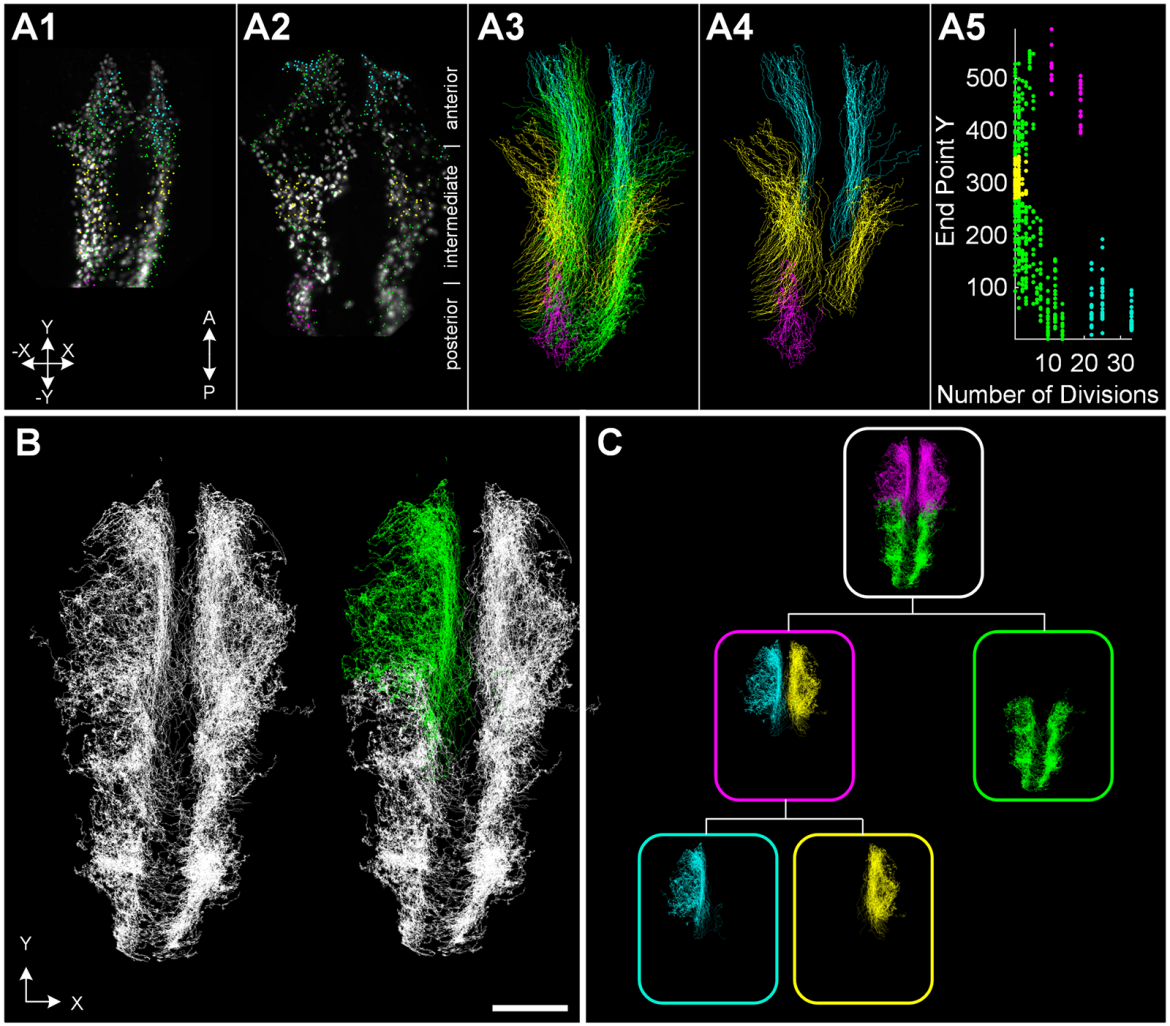
Interactive selection possibilities complement the automatic feature-based group selections. Tracking data from cranial neural crest cells of a zebrafish embryo during 14-20 hpf are shown. (A) All visualization windows allow selecting groups of interest using freehand selection tools. From the left: maximum intensity projection overlay of the raw images and cell centroids for two time points of the neural crest data set at 12.5 hpf and 14 hpf (A1, A2); tracks of all cells in 3D (A3); subset of selected tracks in 3D (A4); scatter plot of track-based features (A5). All visualization windows (A1-A5) are synchronized to obtain consistent selections in all views (see corresponding color-code of panels A1-A5). (B) Exemplary manual selection of a group of interest. Freehand selection tools allow intuitive and interactive selection/deselection of groups of interest. (C) All performed selection steps can be recorded in a hierarchical selection tree view and arbitrary nodes of the tree can be combined to new groups of interest. The hierarchical selection tree view serves as a template to reproduce a particular selection on other data sets including the possibility of refinements to adapt to biological variation of different data sets. The panels show neural crest cells of a zebrafish embryo (12.5 − 28 hpf). Scale bar: 100 *μ*m.

To visualize and analyze specific parts of the embryo in isolation, the user needs the ability to interactively extract groups of interest in any of the data representations, *i.e.*, a possibility to virtually dissect the embryo is required. In addition to the quantitative feature-based description of cell properties, manual selection possibilities in arbitrary visualization windows are required to interact with the data in an intuitive and efficient way. The trajectory database can be globally truncated to a fixed spatial region and by specifying the time interval of interest if the spatiotemporal occurrence of the groups of interest is known in advance. To manually refine the region of interest, we equipped all visualization windows with various selection possibilities. Fig 3A and S4 Video exemplarily illustrate different selection possibilities applied on the neural crest data set using a maximum intensity projection view, a 3D trajectory view and a 2D scatter plot. All data visualization windows are synchronized such that the effect of a selection or deselection is instantaneously updated in all visualization windows (see consistent color code for selected groups in Fig 3A and S4 Video). A freehand tool allows selecting and deselecting single cells at a specific point in time or entire 3D cell tracks from an arbitrary perspective in any of the views (Fig 3B, S4 Video). For increased objectivity, it is possible to perform the selection based on any of the precomputed quantitative features using a 2D scatter plot visualization or by using automatic methods such as clustering (S4 Video).

To keep track of different selections and operations applied on the data, the results of all intermediate analysis steps can be recorded and visualized in a hierarchical selection tree structure as demonstrated in Fig 3C and S4 Video for the neural crest data set. As for the other visualizations, all windows are connected to each other and modifications are automatically propagated through the whole tree. To be able to perform multiple analysis tasks simultaneously, operations can be selectively applied on specific branches of the tree, *e.g.*, to separately focus on anterior/posterior or left/right parts of the embryo as shown in Fig 3C. Besides visualizing intermediate results, all performed steps can be interactively modified and it is possible to combine different nodes to obtain a joint selection. The interactive visual tree structure tool provides a complete overview of the whole analysis pipeline and allows users to concentrate only on subset of the data by interactively choosing the desired tree branch. Moreover, the interactive visual tree structure serves as a template of the complete analysis pipeline and helps to efficiently reproduce selections on another embryo to extract the same information across different data sets. The comprehensive selection possibilities allow one to virtually dissect an entire embryo, to quantitatively analyze a subset of the data and to perform analyses like retrospective cell fate mapping by propagating the cell group associations back in time [61].

### Handling fragmented cell tracking data

Even though fluorescent dyes and microscopy techniques are constantly evolving, automatic approaches to detect, segment and track cell nuclei are still error-prone and it is almost impossible to obtain error-free lineages. Especially in highly complex biological data sets with high cell density, inhomogeneous expression of fluorescent markers, limited spatial or temporal image resolution, a lot of tracking errors may occur. To cope with potentially erroneous tracking data, a subsequent manual curation step is often inevitable in order to obtain longer tracks that ideally cover the entire experimental duration. Particularly, the analysis of global spatiotemporal characteristics of the moving cells or cell lineage analyses rely on the availability of complete tracks.

Based on our visualization framework, we implemented a guided track correction tool that can either be applied on selected groups of tracks or on the entire data set (S2A Fig, S5 and S6 Videos). Possible successor and predecessor tracks are automatically identified based on spatial distance and additional features such as fluorescence intensity difference of potential linking candidates (S2 Note). The measure can be used as an automatic curation heuristic that links fragmented tracks if the distance is below a user-defined threshold. Moreover, the distance measure is used to identify the most likely linking candidates during manual tracking, to efficiently guide the user through the manual curation process with minimal effort (S2 Note, S4 Fig). Different correction modes such as depth-first and breadth-first in both temporal directions help to focus only on the specific corrections required for a particular analysis task or the current group of interest. Moreover, the corrections are supported by interactive maximum intensity projections along all major axes and 3D volume rendering views that focus on the region of interest required for the current correction task. The region of interest used for the 3D volume rendering can be interactively adjusted, to either analyze the cells in the global context or to focus only on a few cells that are required to resolve the respective linking decisions (S5 Video). In cases where only a few representative tracks are corrected manually, the remaining track fragments can be automatically assigned to the groups of interest based on a majority voting of validated tracks in the spatial proximity (S2 Note).

## Results

### Interactive separation of hypoblast and epiblast cells in zebrafish

**Fig 4:**
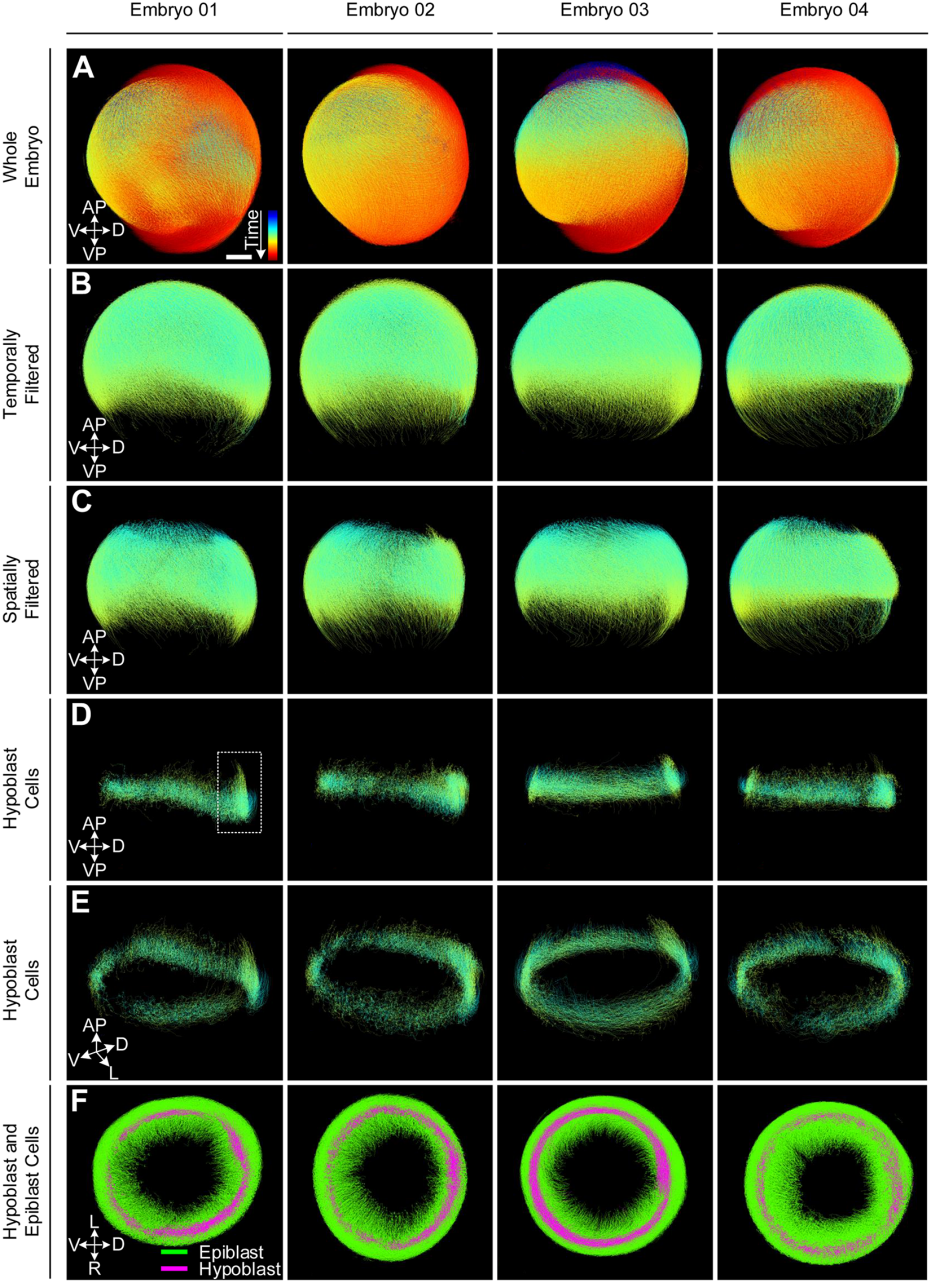
Steps that were performed for extracting hypoblast cells in four different wild-type zebrafish embryos with developmental time ranging from 2–14 hpf (A). The knowledge discovery process was designed interactively on Embryo 01. First, the embryo was filtered temporally (5–7.25 hpf) and spatially (region around the blastoderm margin) to focus on the region of interest (B, C). The two groups of cells were separated using a feature-based clustering approach (D-F). The whole analysis pipeline was then applied to Embryos 02-04 resulting in the extraction of the same internalizing cells in all embryos. The color code in panels (A-E) indicates time from 2–14 hpf and the group association to hypoblast (magenta) or epiblast (green) in panel (F).

To demonstrate the capabilities of the introduced framework, we analyze the first major gastrulation event in zebrafish development, namely the involution of cells at the germ ring margin at about 5.5 hours post fertilization (hpf) [62]. Therefore, we interactively separate hypoblast (involuting) and epiblast (non-involuting) cells in trajectory data of four wild-type zebrafish embryos in an efficient, intuitive and reproducible way. The individual steps performed to separate the two groups of interest are summarized in Fig 4. Starting with the whole embryo data sets (Fig 4A), we cropped a time range of 5 ‒ 7.25 hpf to cover the initial gastrula period (Fig 4B). Furthermore, to focus on the region where involution happens, the embryos were spatially filtered to a region containing the germ ring margin with sufficient coverage of the surrounding tissue towards the animal and the vegetal pole (Fig 4C). The involution happening at the germ ring margin causes the tracks of hypoblast cells to be U-shaped, *i.e.*, their effective displacement is much lower than the integrated path they travel during the gastrula phase (Fig 2E). Using this criterion, we automatically identify two clusters of tracks that were longer than 70% of the total time span and assign all remaining shorter tracks to one of these two classes based on the most common class memberships of labeled tracks in their spatial proximity (Fig 4B–4F, S2 Note, S3 Video). Extracted hypoblast cells are shown in Fig 4D and 4E and the shield region appears as a region of increased cell density at the future dorsal side of the embryo (highlighted in Fig 4D). The color-coded visualizations of both cell groups shown in Fig 4F qualitatively illustrate the localization of both groups within the embryo. The selection pipeline was developed on the first embryo and subsequently reproduced on all other embryos. The possibility to interactively refine all selection steps allowed us to cope with natural variability of the data sets and to easily adapt parameter settings appropriately. Visual comparison of the four columns in Fig 4 indicates that the extracted groups are highly similar.

### Quantitative analysis of tissue deformation during zebrafish gastrulation

To quantify the movement behavior of the separated epiblast and hypoblast cells, we use the mechanical deformation features proposed in [35]. For mathematical details on the feature computations, we refer the reader to the original publication. All features are based on analyzing local deformations of groups of cells with respect to a reference cell in the center of the respective region. Before calculating the mechanical deformation features, we applied a Butterworth low-pass filter with an order of two and a normalized cutoff frequency of 0.01 that preserved the global characteristics of the tracks while efficiently suppressing local noise [63].

**Fig 5:**
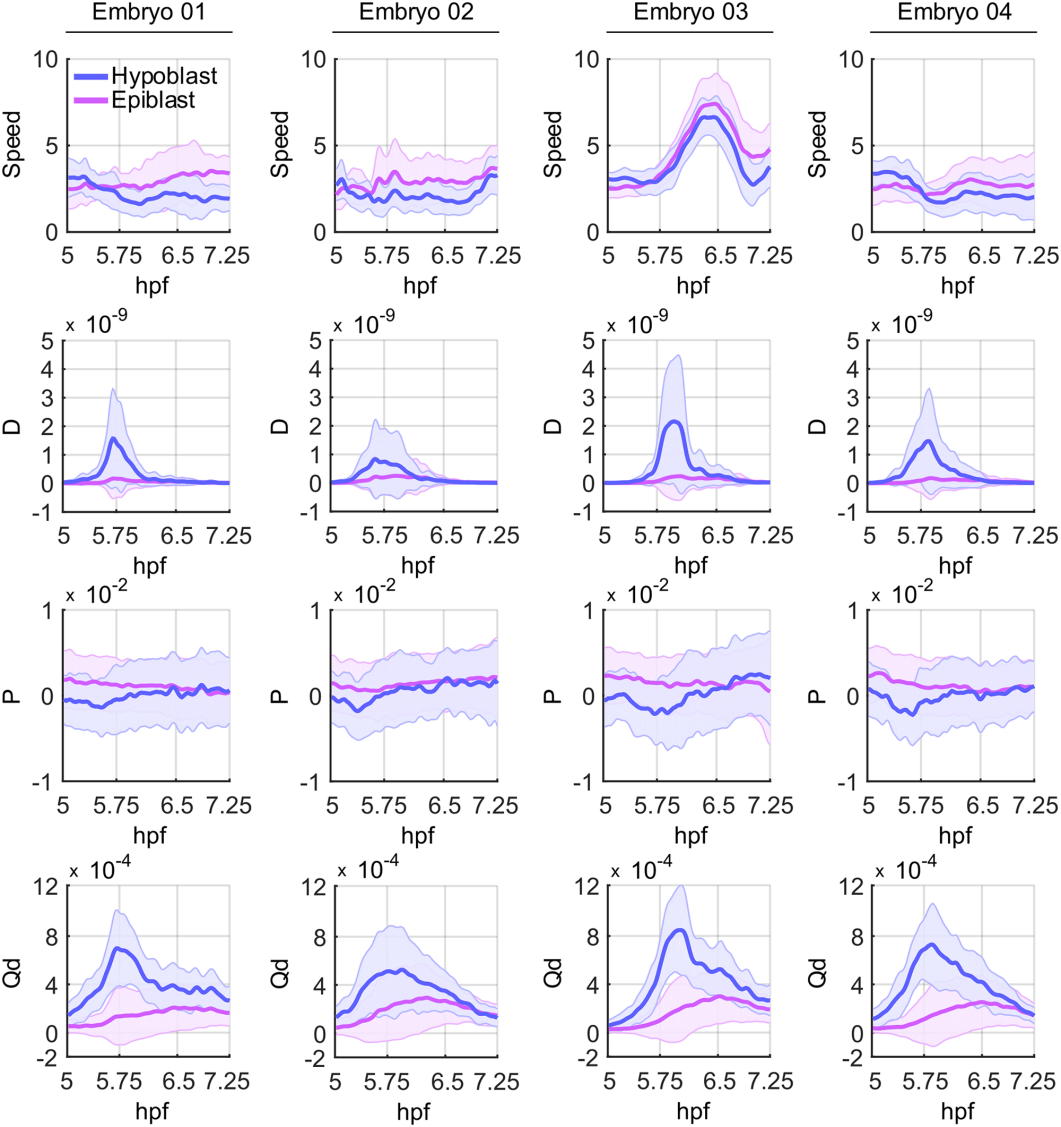
Quantitative comparison of selected tissue deformation features recently published by [35] measured for hypoblast (blue) and epiblast (magenta) cells. Each column contains the results obtained on one of four wild-type zebrafish embryos in a time interval spanning early gastrulation from 5 − 7.25 hpf. The selected features comprise speed, the rotation discriminant *D*, the volume change rate denoted by *P* and the distortion rate *Q*_*d*_ as described in the main text. Note that Embryo 03 physically moved during image acquisition, which caused the increased total speed. Despite this global speed difference, all other quantitative features were nicely captured and revealed comparable patterns among all analyzed embryos.

In Fig 5 and S3 Fig, the quantitative features obtained for hypoblast and epiblast are plotted. We analyzed the speed of each cell (magnitude of the velocity vector), the volume change rate denoted by *P* (values larger/smaller than zero indicate expansion/compression of the tissue), the rotation discriminant *D* (values larger than zero indicate rotation around a reference centroid) and the distortion rate *Q*_*d*_ (indicates that objects contained within a small neighborhood change their relative positions while the surrounding volume remains constant).

At 5 hpf, the hypoblast cells move slightly faster than the epiblast cells. This behavior changes as soon as the involution of the hypoblast starts, *i.e.*, cells located at the interior side of the germ ring change direction and move towards the animal pole of the embryo. In addition, the rotation discriminant *D* peaks between 5.5 hpf and 5.9 hpf, indicating an increased rotational movement at the germ band margin. By contrast, both the speed and the rotation discriminant of the epiblast cells remain more or less constant throughout the analyzed time interval. As one would expect, the volume change rate *P* is larger than zero for the epiblast cells indicating extension movements of this cell group. The time course of *P* measured for the hypoblast cells remains largely around zero with a short negative phase right before the rotation discriminant peaks. Thus, the hypoblast cells seem to temporarily compress at the germ ring margin before involution starts. At 7.25 hpf, the *P* time courses of hypoblast and epiblast converge on each other and remain small but positive, suggesting similar extension movements in both groups of cells. The increased *Q*_*d*_ values of the hypoblast cells peaking at 5.75 hpf may imply that many of those cells change their neighbors without changing the enclosing volume, *i.e.*, suggesting an increased amount of cell intercalations occurring in the hypoblast group. However, this has yet to be confirmed by further analyses, as these quantitative features only provide an initial indicator for this hypothesis.

We note that Embryo 03 physically moved during image acquisition and, thus, the plots for the speed feature depicted in Fig 5 and S3 Fig are globally affected. We intentionally left these panels for demonstration purposes, as these subtle movements were not identified during visual inspection of the maximum intensity projections. Of course, automatic alignment of the data sets before the actual feature extraction would easily get rid of such global movements of the specimens. Nevertheless, only the speed feature was affected by this global movement of Embryo 03. The qualitative behavior of the remaining features is in accordance with the other three wild-type embryos.

### Reconstructing trajectories of precursor cells of the olfactory epithelium in zebrafish

**Fig 6:**
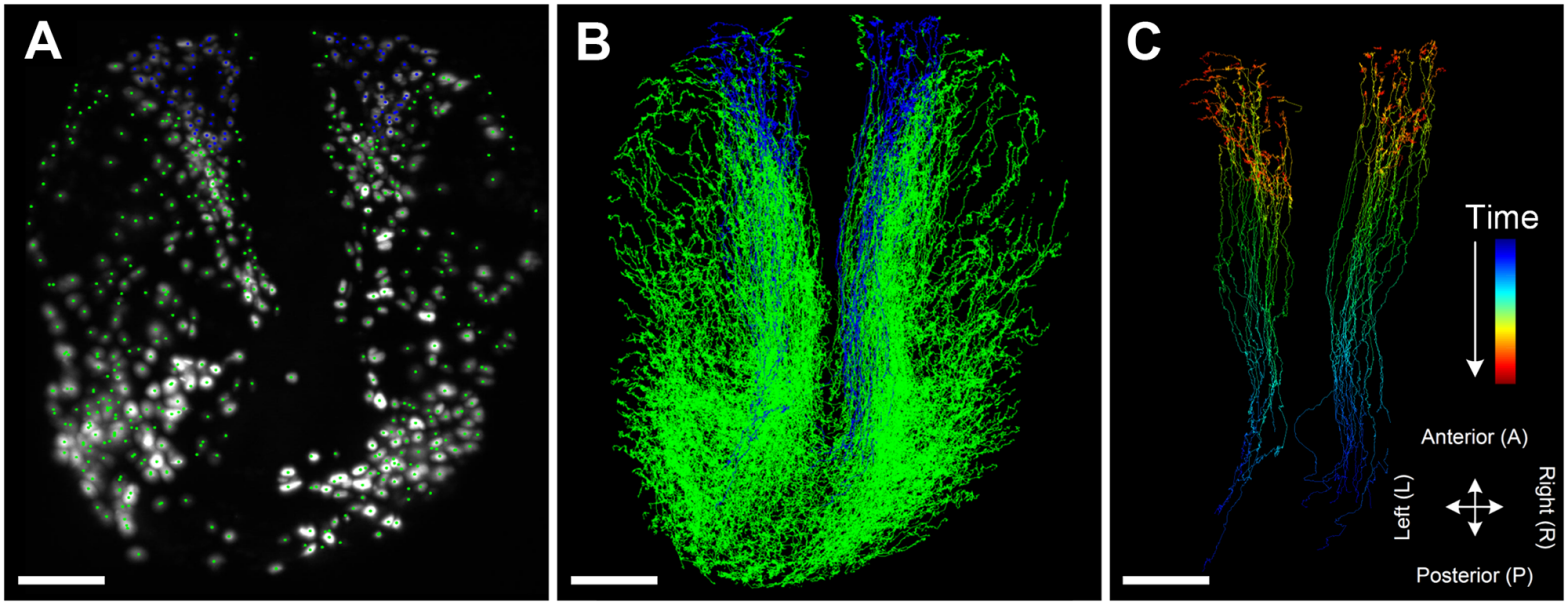
Interactive tracking correction of neural crest cells of a zebrafish embryo. (A) To analyze the precursors of the olfactory epithelium, a particular subgroup of neural crest cells of a zebrafish embryo, a set of about 100 cells was interactively selected at 23 hpf (blue). The corrected tracks are shown in the global context (B) as well as in isolation using time for coloring (C). Scale bar: 100 *μ*m.

Fig 6 and S6 Video show a typical workflow and the results of the track correction steps. A set of about 100 precursor cells of the olfactory epithelium were selected at 23 hpf in the neural crest cell data set (blue selection in Fig 6A). The tracks were interactively corrected using a depth-first search strategy based on the predefined selection of cells of interest. A recent study in zebrafish showed that neural crest cells provide major contribution to microvillous sensory neurons in the olfactory epithelium [64]. The authors elegantly demonstrated the contribution of neural crest cells by photoconversion-based fate mapping and time lapse imaging, however, the origin and their migration path remained not investigated. Here, the trajectories of the olfactory neural crest cells revealed by our retrospective tracking (Fig 6, S6 Video) show bilateral origin of the neural crest cells in the olfactory placode and high directionality toward the anterior direction. The latter high directionality is consistent with the *chase-and-run* behavior of neural crest cells toward olfactory placodal cells [65].

## Availability and future directions

The open-source MATLAB toolbox SciXMiner and the new trajectory analysis framework EmbryoMiner presented in this contribution can be downloaded from https://sourceforge.net/projects/scixminer. We provide a quickstart guide containing installation instructions and details on how to test the application in S3 Note. A general introduction to SciXMiner can be found in [59] and a detailed documentation of the individual components of EmbryoMiner are shipped with the extension package. Moreover, we provide sample data that can be used to understand the presented components of the framework including all visualization modules, selection possibilities, application of data mining methods and data import.

In future releases, we plan to further facilitate the transfer of the analysis pipelines to new embryos, *e.g.*, by automatically detecting and registering corresponding anatomical regions of interest or by training a classifier on the first data set and by automatically classifying all tracks of the remaining data sets automatically using the pretrained classifier. Moreover, we plan to add more sophisticated curation heuristics that potentially can go back to the original image data to resolve ambiguous matches or to add missing links automatically.

## Supporting information

S1 Note Image acquisition, segmentation and tracking in brief. (PDF)

S2 Note Materials and methods. (PDF)

S3 Note Quickstart guide. (PDF)

S1 Fig Preprocessing steps to automatically detect, segment and track fluorescently labeled nuclei in light-sheet microscopy images of a zebrafish embryo. (A) Maximum intensity projection of a single time point of an entire zebrafish embryo at early somitogenesis stages [11, 66]. (B) Maximum intensity projection of a 3D light-sheet microscopy image with superimposed centroids of detected cell nuclei in red. (C) Volume rendering of segmented cell nuclei using a random color code. (D) Exemplary movement paths for 25 arbitrarily selected neural crest nuclei of a zebrafish embryo that were automatically tracked using a nearest neighbor algorithm [58, 59]. Dorsal view of the anterior half of the embryo (anterior up). All data sets were aligned prior to the analysis, to obtain a common reference orientation. (E) Whole-embryo data sets were oriented such that the animal-vegetal axis was aligned with the y-axis (animal pole on the positive y-axis) and the dorsoventral axis was aligned with the x-axis (dorsal part on the positive x-axis). (F) The embryos highlighting neural crest cells were oriented such that the anteroposterior axis formed a left-right symmetry axis with the head region on the positive y-axis (dorsal view, anterior up). Circled regions in (F) indicate the positions of the prospective eyes and the olfactory epithelium of the embryo, respectively. The green rectangle in panels (A) and (E) indicates the neural crest cell region visualized in (F). Panels (A)-(D) were adapted from [67] and panel (E) was adapted from [11]. Scale bar: 100 *μ*m.
(TIF)

S2 Fig Interactive and semi-automatic strategies to correct and analyze fragmented cell tracks. (A) Erroneous tracks can be interactively repaired to obtain a set of corrected full-length tracks. The curation mode features interactive maximum intensity projections and 3D volume rendering with superimposed cell tracks. Moreover, link candidate predictions and different curation modes like breadth-first or depth-first curation strategies minimize the required manual correction effort. (B-F) As an alternative to manually correcting the entire tracking database, we propose to select only a subset of sufficiently long tracks (B, *e.g.*, select only tracks spanning over 70% of the time interval of interest), to perform the region of interest selection on these corrected tracks (C-E) and to finally assign all unlabeled tracks to the predominating group of their spatiotemporally nearest neighbors (F). Panel (A) shows a C. elegans embryo of the Cell Tracking Challenge that was imported to EmbryoMiner (data set by the Waterston lab, The George Washington University, Washington D.C., USA) [24, 36] and panels (B-F) show cropped regions of a whole-embryo zebrafish data set (S2 Note).
(TIF)

S3 Fig Quantitative comparison of selected tissue deformation measured for all hypoblast cells of all embryos (first column), all epiblast cells (second column), all cells present in selected time window of 5 − 7.25 hpf (third column) and the spatially cropped data selection (fourth column). The extracted features are identical to the ones depicted in Fig 5 but all embryos are shown in a single plot for easier comparison. Except for the speed feature of Embryo 03 that was affected by the movement during image acquisition, all other quantitative features were nicely captured and revealed comparable patterns among all analyzed embryos and groups. The group selections in the last two columns show that properly selected regions are crucial to avoid the superposition of different movement behaviors.
(TIF)

S4 Fig Quantitative assessment of the manual curation module. (A) Distribution of the selected correct link candidate, where Candidates 1-3 are the automatic suggestions computed by the framework and Candidate 4 indicates that the user selected an alternative link candidate to be correct. (B) Box plots of the distance in pixel of the selected link candidate. (C) Box plot of the time in seconds that was required by the manual annotator to perform the decision. For box plots in (B) and (C), the median is indicated by red bar and the 25% and 75% quantiles are indicated by the lower and upper extents of the box, respectively. (D) Scatter plot of time in seconds versus the distance of the linked candidate. Color and symbols indicate, which candidate has been selected. (E) Selected trajectories of the neural crest data set before (left) and after the manual curation (right). Time is indicated by the color-code from blue to red.
(TIF)

S1 Video Importers for the tracking algorithms implemented in the TGMM, BioEmergences, TrackMate or Cell Tracking Challenge format. These importers allow applying the proposed interactive analysis approaches on tracking results obtained by other existing pipelines. The video demonstrates how, *e.g.*, data extracted with the BioEmergences workflow [33] can be imported and analyzed using the presented framework.
(MP4)

S2 Video Overview of the provided visualizations to interactively analyze 3D trajectory data. The visualized sample data shows neural crest cell development of a zebrafish embryo from 12.5 − 28.0 hpf as a 3D trajectory rendering and an overlay on a 2D maximum intensity projection. The final part of the video shows an interactive 3D volume rendering visualization with superimposed detections of a C. elegans embryo that was imported to EmbryoMiner (Cell Tracking Challenge data set by the Waterston lab, The George Washington University, Washington D.C., USA, [24, 36]).
(MP4)

S3 Video Exemplary trajectory feature visualization. Single features, time series and selections can be used for colorization of the visualized trajectories. The video shows the density (calculated as the number of neighbors within a fixed sphere of 40 *μ*m surrounding each nucleus). Furthermore, we show the hypoblast (magenta) and epiblast (green) cells that were automatically identified.
(MP4)

S4 Video Overview of the selection possibilities provided by the framework. Selections can be performed on trajectory level in 3D, at a specific point in time in a 2D projection or based on trajectory features (manual selection in a scatter plot or using feature-based clustering and classification). All views and selections are synchronized and a hierarchical selection tree allows combining arbitrary groups and to apply a selection pipeline to other data sets.
(MP4)

S5 Video Demonstration of the trajectory curation framework. Erroneous tracks can be interactively repaired to obtain a set of corrected full-length tracks using maximum intensity projections and 3D volume rendering of the raw image data with superimposed detections and tracks. The curation process is assisted using link candidate predictions and different curation modes like a breadth-first or a depth-first curation strategies. In addition to the full data set view, it is possible to shrink the visualized region of interest to minimize distraction from the current link candidate by the surrounding tissue. The video shows a C. elegans embryo of the Cell Tracking Challenge that was imported to EmbryoMiner (data set by the Waterston lab, The George Washington University, Washington D.C., USA) [24, 36].
(MP4)

S6 Video Application of the trajectory curation framework to precursor cells of the olfactory epithelium in zebrafish. For demonstration purposes, we selected about 100 precursor cells of the olfactory epithelium at 23 hpf and corrected the corresponding tracks using a depth-first strategy. The video illustrates the selection of the group of interest, some exemplary linking actions and the final results of the curation.
(MP4)
