# Supplementary material for: EmbryoMiner: A new framework for interactive knowledge discovery in large-scale cell tracking data of developing embryos
Source: PLoS Comput Biol. 2018 Apr 19;14(4):e1006128. doi: 10.1371/journal.pcbi.1006128 (PMC5929571; doi:10.1371/journal.pcbi.1006128)
Supplement: S1 Note — (PDF) [file pcbi.1006128.s001.pdf]

# EmbryoMiner: A new framework for interactive knowledge discovery in large-scale cell tracking data of developing embryos

Benjamin Schott[1,*], Manuel Traub[1], Cornelia Schlagenhauf[1], Masanari Takamiya[2], Thomas Antritter[1], Andreas Bartschat[1], Katharina Löffler[1], Denis Blessing[1], Jens C. Otte[2], Andrei Y. Kobitski[3], G. Ulrich Nienhaus[2,3,4,5], Uwe Strähle[2], Ralf Mikut[1], Johannes Stegmaier[1,6,*]

**1** Institute for Automation and Applied Informatics, Karlsruhe Institute of Technology, Karlsruhe, Germany
**2** Institute of Toxicology and Genetics, Karlsruhe Institute of Technology, Karlsruhe, Germany
**3** Institute of Applied Physics, Karlsruhe Institute of Technology, Karlsruhe, Germany
**4** Institute of Nanotechnology, Karlsruhe Institute of Technology, Karlsruhe, Germany
**5** Department of Physics, University of Illinois at Urbana-Champaign, Urbana, Il, USA
**6** Institute of Imaging and Computer Vision, RWTH Aachen University, Aachen, Germany

* benjamin.schott@kit.edu or johannes.stegmaier@lfb.rwth-aachen.de

## S1 Note: Image acquisition, segmentation and tracking in brief

Wild-type zebrafish embryos expressing an ubiquitous fluorescent marker in their cellular nuclei *Tg(h2afva:h2afva-GFP) kca66Tg* and a neural crest reporter line *Tg(-7.2sox10:h2afva-EosFP)* were imaged with our custom-made light-sheet microscope (S1 Fig A, S2 Note) for the analysis of cellular dynamics during early gastrulation on the whole-embryo level and post-gastrulation stages (11-28 hpf) of neural crest cells, respectively. Fluorescently labeled nuclei were detected separately in each of the acquired 3D images (S1 Fig B-C, S2 Note) and subsequently tracked to obtain the spatiotemporal movement trajectories for all detected objects (S1 Fig D, S2 Note). Finally, the resulting cell tracks were transformed to a standard orientation as described in [1]. The whole embryo data sets were temporally synchronized at the 256-cell stage and the orientation was performed such that the animal-vegetal axis extended from the positive y-axis to the negative y-axis. Furthermore, the prospective dorsoventral axis of the embryo was aligned with the x-axis with the prospective ventral side on the negative x-axis and the prospective dorsal side on the positive x-axis (S1 Fig E). The neural crest data sets were oriented such that the anteroposterior axis was aligned with the y-axis with anterior and posterior sides lying on the positive and the negative parts of the y-axis, respectively. The left-right axis extended from negative to positive x-axis (S1 Fig F). These oriented cell movement databases were then used for all further analyses and demonstrations presented throughout this paper. See S2 Note for the ethics statement and further details on the image acquisition, image analysis and cell tracking.

## References

1. Kobitski AY, Otte JC, Takamiya M, Schäfer B, Mertes J, Stegmaier J, et al. An Ensemble-averaged, Cell Density-based Digital Model of Zebrafish Embryo Development Derived from Light-sheet Microscopy Data with Single-cell Resolution. Scientific Reports. 2015;5(8601):1–10.
