# Supplementary material for: EmbryoMiner: A new framework for interactive knowledge discovery in large-scale cell tracking data of developing embryos
Source: PLoS Comput Biol. 2018 Apr 19;14(4):e1006128. doi: 10.1371/journal.pcbi.1006128 (PMC5929571; doi:10.1371/journal.pcbi.1006128)
Supplement: S2 Note — (PDF) [file pcbi.1006128.s002.pdf]

EmbryoMiner: A new framework for interactive knowledge discovery in large-scale cell tracking data of developing embryos

Benjamin Schott[1,*], Manuel Traub[1], Cornelia Schlagenhauf[1], Masanari Takamiya[2], Thomas Antritter[1], Andreas Bartschat[1], Katharina Löffler[1], Denis Blessing[1], Jens C. Otte[2], Andrei Y. Kobitski[3], G. Ulrich Nienhaus[2,3,4,5], Uwe Strähle[2], Ralf Mikut[1], Johannes Stegmaier[1,6,*]

1 Institute for Automation and Applied Informatics, Karlsruhe Institute of Technology, Karlsruhe, Germany
2 Institute of Toxicology and Genetics, Karlsruhe Institute of Technology, Karlsruhe, Germany
3 Institute of Applied Physics, Karlsruhe Institute of Technology, Karlsruhe, Germany
4 Institute of Nanotechnology, Karlsruhe Institute of Technology, Karlsruhe, Germany
5 Department of Physics, University of Illinois at Urbana-Champaign, Urbana, Il, USA
6 Institute of Imaging and Computer Vision, RWTH Aachen University, Aachen, Germany

* benjamin.schott@kit.edu or johannes.stegmaier@lfb.rwth-aachen.de

S2 Note: Materials and methods

Ethics statement

Zebrafish (*Danio rerio*) wild-type embryos expressing a fluorescent marker in the cell nucleus *Tg(h2afva:h2afva-GFP) kca66Tg* and neural crest reporter line *Tg(-7.2sox10:h2afva-Eos)* were used in this study. Zebrafish husbandry and experimental procedures were performed in accordance with German animal protection regulations (Regierungspräsidium Karlsruhe, Germany, AZ35-9185.81/G-137/10).

Sample preparation, image acquisition and data storage

For details on the data preparation, image acquisition and data storage see [1]. To reduce background fluorescence the chorions were manually removed and the zebrafish embryos were mounted in vertically oriented fluorinated ethylene propylene (FEP) tubes using 0.1% agarose to minimize effects due to mechanical hindrance. Afterwards, the embryos were positioned in the sample chamber at 26.5°C. Images of the embryos were taken on a custom-made digital scanned laser light-sheet microscope (DSLM) [1]. To achieve continuous imaging at high speed, the data acquisition process was parallelized wherever possible. The acquisition time and resolution is comparable to results achieved by simultaneous multiview imaging with two detection objectives and two cameras. Starting at the 8-64 cell stage, the 3D image stacks were taken continuously for 16 h. Fluorescence was excited with lasers, emitting at 488 nm for GFP and 561 nm for mCherry. A water dipping objective (CFI-75 LWD 163/0.8w, Nikon GmbH, Düsseldorf, Germany) was used to collect the fluorescent emission with an sCMOS camera (Neo, Andor, Belfast, UK). For parallel processing of the DSLM images, the raw images were transferred to the Large Scale Data Facility (LSDF) at the

Steinbuch Center for Computing that features petabytes of storage space and a computing cluster for accelerated processing [2]. To match the developmental stages described by Kimmel *et al.* at a temperature of 28.5°C [3], the developmental time axis was scaled accordingly. Therefore, the 256-cell stage (2.5 hpf) was selected as a starting point and the measurement time was down-scaled by 10% as the experiments were performed at 26.5°C. Images were acquired every 60 and 20 s for early gastrulation and post gastrulation neural crest data sets, respectively.

Extracting tracks from 3D+t image data

Fluorescently labeled nuclei in the 3D images were detected using a Laplacian-of-Gaussian Scale Space Maximum Projection (LoGSSMP) approach as described in [4]. Images were filtered using a 3D Laplacian-of-Gaussian (LoG) filter with standard deviations that matched the size range of the nuclei (σ_{min}=5, σ_{max}=8). For each voxel, we identified the maximum value obtained by the LoG-filter, to generate the scale-space maximum projection image. We then detected the centroids of cellular nuclei in the LoGSSMP as non-strict local maxima [5]. Redundant detections on intensity plateaus within a radius of 3 pixels (px) were then combined to a single detection and for each detection we calculated the mean intensity within a radius of 2 px to get a more robust intensity estimate of the seed point.

The seed detection step basically extracts all local maxima that are present in the LoGSSMP image. To suppress false positive detections in background regions, a global intensity threshold was applied on the mean intensity of each seed point, *i.e.*, seed points with an intensity below this threshold were rejected. The threshold was manually adjusted using an interactive graphical user interface showing the filtered detections on the maximum intensity projections along the X, Y and Z direction as described in [5, 6]. The optimal registration was manually identified using a custom-made graphical user interface. As the rotation of the views was known to be 180°, only the translation had to be estimated which could be easily achieved using the GUI. We then used ParaView to validate the registration results in 3D for a selection of representative time points. Subsequently, we used the centroid of all detections at each time point as the cutoff criterion for fusing the two views, i.e., only the detections of each image in regions with good image quality were used for the final data sets.

The tracking was directly performed on filtered, registered and fused seed points and a segmentation step as described in [4] was skipped here for faster processing. However, if additional features such as the actual size of the objects, average fluorescence intensities or shape properties are of interest, a subsequent seeded segmentation approach can be added and the centroids of identified segments can be used instead of the seed points. We used a nearest neighbor tracking approach implemented in SciXMiner by searching for the spatially nearest neighbor starting at the last frame backwards to the first frame. A maximum distance for allowed associations was estimated based on the distance of the local neighbors, *i.e.*, linking was only considered unambiguous if the distance traveled by an object between two frames was less than half the distance to its local neighbors in the current frame. This provided a fast and easy solution that yielded a set of reliable tracks stored as a SciXMiner-compatible time-series project, *i.e.*, a 3D matrix of size $N \times K \times F$ with N objects, K time points and F features that can be manipulated using basic MATLAB operations or via the graphical user interface. To obtain error-free full-length trajectories including correct division events, however, manual corrections may be required. It should be noted that the tracking algorithm itself was not the focus of the present contribution and we thus provide additional importers for other tracking algorithms. All remaining steps described in this paper can be equally performed with tracking data from a different source.

Spatial alignment of the whole-embryo data sets

In addition to the temporal synchronization of the data sets at the 256-cell stage, the four wild-type embryos used for the comparative analysis of epiblast and hypoblast cells were spatially transformed to a standard orientation as visually illustrated in S1 Fig E. In brief, the 16 hpf stage was used to fit a sphere to the spherical embryo and to move the center of the estimated 3D sphere to the origin. Subsequently, each data set was rotated, such that the animal-vegetal axis extended from the positive to the negative y-axis. Similarly, the prospective dorsoventral axis was aligned with the x-axis, extending from the positive to the negative x-axis. The transformation was then applied to all preceding time points. To compensate for potential movements of the embryo during development a set of rules was used to stabilize the orientation, such that the epiboly direction consistently ran along the y-axis and that cells were evenly distributed with respect to the xy-plane. For further details on the sample alignment, see [1]. The final analyses presented in the results section were performed using these spatially aligned and temporally synchronized data sets.

Semi-automatic correction of fragmented tracks

To obtain error-free tracks in cases where automatic tracking approaches fail, we developed a data curation framework that automatically computes potential link candidates and offers the possibility to manually correct erroneous links.

On the basis of the local neighborhood at each of the start points of a fragmented track (*i.e.*, positions where a track fragment was not successfully linked so far), we identified potential predecessors and assessed the quality of the linking. Under the assumption that most links in the local neighborhood are correct, we identify distributions of predefined features and calculate the probability of linking a potential predecessor based on these distributions. In the current implementation, the spatial distance between consecutive cell positions as well as the intensity difference between the segmented cell nuclei were used and it is straightforward to include additional features. Potential predecessors are also calculated for multiple consecutive time points to allow skipping a time point in case of a temporary misdetection. We found that allowing to skip maximally three frames provided the best results. The number of neighbor candidates presented to the user as well as the threshold for the quality measure can be parametrized in the SciXMiner GUI depending on the respective scenario. In the manual curation mode, the quality measure is used to visualize the successor and predecessor track fragments with the highest probability to be correct to the user. In one third of the cases, the correct match is among the first three suggestions and the user only needs to identify the best linking candidate visually (S4 Fig A, B). Even if the correct link candidate is not among the suggestions, it usually takes less than 20 seconds to identify the correct link (S4 Fig C, D). Internal linking conflicts, such as linking too many fragments to one predecessor track, are handled automatically. In cases where only a rough overview of the global movement characteristics of a data set might be required, the closest identified link candidate can be used to automatically connect all fragmented tracks with a quality measure higher than a predefined threshold. It should be noted, though, that this fully automatic approach potentially introduces linking errors and it is thus not suitable for analyses where accurate single-cell tracking is required (S4 Fig A). In these cases, it is inevitable to stick to the interactive manual curation module of the framework.

Based on the corrected tracking data that ideally span the entire experimental duration (S4 Fig E), experiments like retrospective cell fate mapping become feasible, *i.e.*, structures of interest can be selected at an arbitrary point in time and it is possible to investigate the movement patterns of all precursors and descendants of the group of

interest both qualitatively using the visualization modules and quantitatively based on trajectory features.

Automatic group assignments of fragmented tracks

As an alternative to laborious manual correction of fragmented tracks, we additionally developed a strategy that can circumvent the problems arising with fragmented tracking data. Initially, a time interval of interest has to be defined and we select only tracks that sufficiently cover the desired time span, *i.e.*, short fragments of the tracks are excluded from the initial analysis (S2 Fig B). These sufficiently long tracks serve as representatives for the different groups contained in the data set (S2 Fig B-F). Next, all group selection strategies can be applied to this subset of sufficiently long tracks that allow analyses over the entire time interval of interest (S2 Fig E). To assign the remaining unlabeled track fragments (yellow track in S2 Fig E) to the identified groups of interest, we compare each track fragment to its local neighborhood and identify the spatiotemporally nearest neighbors (S2 Fig E). For the results presented here, track fragments that covering same time interval within a confined spatial region around each track (*e.g.*, a cube or sphere surrounding each track fragment) were considered to be neighbors. Next, we computed the Euclidean distance between all points of a considered track fragment and all points of the remaining track fragments to obtain the pairwise distances. The group membership of each unlabeled track fragment is then determined by the occurrence frequency of extracted groups within the local neighborhood (S2 Fig F). Throughout this paper we used tracks that were covering the analyzed time window by at least 70% for the identification of groups of interest and allocated all remaining track fragments using the $k = 15$ nearest neighbors in the set of representative tracks after the groups of interest have been identified.

It is also possible to combine this automatic assignment with the manual correction mode by initially correcting a set of representative tracks manually, by subsequently using these tracks to identify groups of interest and by finally assigning all remaining track fragments to the group of the spatiotemporally closest neighbor (S5 Video). Trajectory features that require tracks to span the entire time interval are still not feasible with this track fragment assignments. However, measurements like local tissue deformation, migration speed and cell density can be measured reliably for the identified groups (Fig. 4 and S3 Fig).

References

1. Kobitski AY, Otte JC, Takamiya M, Schäfer B, Mertes J, Stegmaier J, et al. An Ensemble-averaged, Cell Density-based Digital Model of Zebrafish Embryo Development Derived from Light-sheet Microscopy Data with Single-cell Resolution. Scientific Reports. 2015;5(8601):1–10.

2. Garcia AO, Bourov S, Hammad A, van Wezel J, Neumair B, Streit A, et al. The Large Scale Data Facility: Data Intensive Computing for Scientific Experiments. In: Proc., IEEE International Symposium on Parallel and Distributed Processing Workshops and Phd Forum. IEEE; 2011. p. 1467–1474.

3. Kimmel CB, Ballard WW, Kimmel SR, Ullmann B, Schilling TF. Stages of Embryonic Development of the Zebrafish. Developmental Dynamics. 1995;203(3):253–310.

4. Stegmaier J, Otte JC, Kobitski A, Bartschat A, Garcia A, Nienhaus GU, et al. Fast Segmentation of Stained Nuclei in Terabyte-Scale, Time Resolved 3D Microscopy Image Stacks. PLOS ONE. 2014;9(2):e90036.

5. Stegmaier J, Mikut R. Fuzzy-based Propagation of Prior Knowledge to Improve Large-Scale Image Analysis Pipelines. PLOS ONE. 2017;12(11):e0187535.

6. Stegmaier J, Arz J, Schott B, Otte JC, Kobitski A, Nienhaus GU, et al. Generating Semi-Synthetic Validation Benchmarks for Embryomics. In: IEEE 13th International Symposium on Biomedical Imaging (ISBI). IEEE; 2016. p. 684–688.
