# Supplementary material for: EmbryoMiner: A new framework for interactive knowledge discovery in large-scale cell tracking data of developing embryos
Source: PLoS Comput Biol. 2018 Apr 19;14(4):e1006128. doi: 10.1371/journal.pcbi.1006128 (PMC5929571; doi:10.1371/journal.pcbi.1006128)
Supplement: S3 Note — (PDF) [file pcbi.1006128.s003.pdf]

EmbryoMiner: A new framework for interactive knowledge discovery in large-scale cell tracking data of developing embryos

Benjamin Schott[1,*], Manuel Traub[1], Cornelia Schlagenhauf[1], Masanari Takamiya[2], Thomas Antritter[1], Andreas Bartschat[1], Katharina Löffler[1], Denis Blessing[1], Jens C. Otte[2], Andrei Y. Kobitski[3], G. Ulrich Nienhaus[2,3,4,5], Uwe Strähle[2], Ralf Mikut[1], Johannes Stegmaier[1,6,*]

1 Institute for Automation and Applied Informatics, Karlsruhe Institute of Technology, Karlsruhe, Germany
2 Institute of Toxicology and Genetics, Karlsruhe Institute of Technology, Karlsruhe, Germany
3 Institute of Applied Physics, Karlsruhe Institute of Technology, Karlsruhe, Germany
4 Institute of Nanotechnology, Karlsruhe Institute of Technology, Karlsruhe, Germany
5 Department of Physics, University of Illinois at Urbana-Champaign, Urbana, Il, USA
6 Institute of Imaging and Computer Vision, RWTH Aachen University, Aachen, Germany

* benjamin.schott@kit.edu or johannes.stegmaier@lfb.rwth-aachen.de

S3 Note: Quickstart guide

As a prerequisite to run EmbryoMiner, make sure MATLAB and SciXMiner are properly installed. The latest release of SciXMiner can be obtained from `https://sourceforge.net/projects/scixminer/` and general information as well as installation instructions for SciXMiner are provided in the following document: `https://arxiv.org/abs/1704.03298`. To install EmbryoMiner and to run the examples, perform the following steps:

1. Download and install the EmbryoMiner toolbox from `https://sourceforge.net/projects/scixminer/files/Extension%20packages/` by extracting the `embryominer.zip` archive to the `application_specials` folder of your SciXMiner installation. This extension package contains all required components of the interactive knowledge discovery framework EmbryoMiner.

2. Open MATLAB and start SciXMiner by typing `scixminer` in the command line window of MATLAB. If you don't see the `EmbryoMiner` entry in the menu bar, open `Extras` → `Choose application specific extension packages ...` and activate the tracking toolbox. After restarting SciXMiner, the tracking toolbox should properly load and everything is ready for running the application examples.

3. Download the application examples from `https://sourceforge.net/projects/scixminer/files/InteractiveKnowledgeDiscovery/` and extract them to a folder of your choice.

4. Return to the SciXMiner GUI and select one of the examples from the `InteraciveKDExamples` folder with the extension `*.batch`. Use the menu entry `File` → `Apply SciXMiner Batch File` and select the example of your choice.

5. The `*.batch` file automatically loads the demo projects as well as the visualization windows. Batch files can be opened in a standard text editing software, in case you're interested in understanding or changing the code.

6. Further information is provided in the readme files associated with each of the examples. The application examples comprise an overview of the visualization possibilities, interactive selection capabilities, application of data mining methods to cell tracking data, track filtering as well as the import of tracking data generated by other tools.

The software was tested and compiled on Microsoft Windows 10 using MATLAB 2017b. Releases for the other major operating systems are planned for the next release.

Notes:

- To get an impression of how to interact with SciXMiner and to get used to its workflow, make sure to have a look at the supplementary items S1 − S5 Video that resemble the application examples provided with the software.
- A detailed description of all functions of the graphical user interface of EmbryoMiner are provided in the file `scixminer_tracking_help.pdf` that is part of the tracking toolbox.
- We tested our framework with entire zebrafish embryos and found that the framework remains highly responsive up to at least 25,000 objects per frame. Thus, all examples presented in this paper could be performed on a usual desktop computer. We note that the responsiveness for larger data sets that include 3D volume rendering may decrease or may require a more powerful workstation with sufficient memory. As most of the analyses are constrained to a specific region of interest, however, performing a spatiotemporal filtering of the data sets prior to the actual analysis can be used to reduce the track amount to a feasible range.
- The seed detection and segmentation presented in the main text was implemented in the open-source software tool XPIWIT [1,2], a platform-independent application that was implemented in C++ on the basis of the Insight Toolkit [3]. XPIWIT is applicable to large-scale 3D images, features a graphical user interface and XML pipelines for processing the data (download and installation instructions available from *https://bitbucket.org/jstegmaier/xpiwit/downloads*). Extracted segments or seed points can be imported and tracked with SciXMiner using the menu item `EmbryoMiner` → `Import / Export / Convert` → `Import (XPIWIT) CSV Files`.
- The menu item `EmbryoMiner` → `Import / Export / Convert` → `Import (XPIWIT) CSV Files` can also be used as a generic importer for CSV files. Each time point is required to have a separate CSV file with a single detection per row. The first row contains the specifiers for each of the columns (separated by semicolon (";"), dot (".") to specify floating point values). The import script assumes that columns 3, 4, 5 contain the x, y, z locations of each object and are named as "xpos", "ypos", "zpos". Empty rows should have the same number of columns and entries should be filled with the "NaN" specifier. If required, provided locations will be automatically tracked using a nearest neighbor tracking algorithm and converted into a SciXMiner project (MATLAB `*.mat` files). The imported project will be saved a level above the folder that contained the CSV files.

- SciXMiner projects can be exported to CSV (one CSV file per time point) via the menu command `EmbryoMiner` → `Import / Export / Convert` → `Export Project as CSV Files`.

- To use the 3D volume viewer feature of the framework, image data sets have to be converted to the `*.mha` format first. We provide an importer script that automatically converts `*.tif` images to the appropriate format in the menu item `EmbryoMiner` → `Import / Export / Convert` → `Convert 3D Tiffs to MHA for Volume Rendering`. The script can also be used to generate maximum intensity projections along all major axes, if enabled in the settings dialog. Once a project is loaded and the VTK Visualization was started properly using `EmbryoMiner` → `VTK Visualization` → `Start/Restart`, 3D visualizations and 2D maximum intensity projections can be added via `EmbryoMiner` → `VTK Visualization` → `Add ... Projection` and by selecting all 3D volume files or the desired maximum intensity projection file. We tested the new 3D viewer with the largest available data sets of the Cell Tracking Challenge (1272 x 603 x 125 px, 8 bit, 50 time points) and the BioEmergences data sets (512 x 512 x 104 px, 8 bit, 360 time points) and scrolling in time was still possible and highly responsive even on a standard desktop workstation (Intel Core i7-6700 CPU @ 3.4GHz, 64GB memory, NVidia Quadro K620 GPU). For even larger images, however, we note that a workstation with a sufficiently powerful GPU might be required. Alternatively, strategies like down-sampling or cropping may be used to maintain the interactivity on less powerful computers.

- The software features importers for the cell tracking algorithms TGMM [4], BioEmergences [5], TrackMate [6] and algorithms that produce output in the Cell Tracking Challenge format [7, 8]. All third-party importers are available in the SciXMiner menu item `EmbryoMiner` → `Import / Export / Convert` → `...`. The procedure is self-explanatory and the window titles of the file open dialogs guide you through the import process.

- If you have any problems or questions related to the trajectory visualization framework, please do not hesitate to write us an email (benjamin.schott@kit.edu or johannes.stegmaier@partner.kit.edu), ideally including a detailed description on how to reproduce the respective error.

References

1. Stegmaier J, Otte JC, Kobitski A, Bartschat A, Garcia A, Nienhaus GU, et al. Fast Segmentation of Stained Nuclei in Terabyte-Scale, Time Resolved 3D Microscopy Image Stacks. PLOS ONE. 2014;9(2):e90036.

2. Bartschat A, Hübner E, Reischl M, Mikut R, Stegmaier J. XPIWIT - An XML Pipeline Wrapper for the Insight Toolkit. Bioinformatics. 2016;32(2):315–317.

3. Ibanez L, Schroeder W, Ng L, Cates J, et al. The ITK Software Guide. Citeseer; 2005.

4. Amat F, Lemon W, Mossing DP, McDole K, Wan Y, Branson K, et al. Fast, Accurate Reconstruction of Cell Lineages from Large-Scale Fluorescence Microscopy Data. Nature Methods. 2014;11(9):951–958.

5. Faure E, Savy T, Rizzi B, Melani C, Stašová O, Fabrèges D, et al. A Workflow to Process 3D+ Time Microscopy Images of Developing Organisms and Reconstruct their Cell Lineage. Nature Communications. 2016;7(8674).

6. Tinevez JY, Perry N, Schindelin J, Hoopes GM, Reynolds GD, Laplantine E, et al. TrackMate: An Open and Extensible Platform for Single-Particle Tracking. Methods. 2016;115:80–90.

7. Maška M, Ulman V, Svoboda D, Matula P, Matula P, Ederra C, et al. A Benchmark for Comparison of Cell Tracking Algorithms. Bioinformatics. 2014;30(11):1609–1617.

8. Ulman V, Maška M, Magnusson KE, Ronneberger O, Haubold C, Harder N, et al. An Objective Comparison of Cell-Tracking Algorithms. Nature Methods. 2017;14:1141.
