# Supplementary figures and images for: EmbryoMiner: A new framework for interactive knowledge discovery in large-scale cell tracking data of developing embryos
Source: PLoS Comput Biol. 2018 Apr 19;14(4):e1006128. doi: 10.1371/journal.pcbi.1006128 (PMC5929571; doi:10.1371/journal.pcbi.1006128)

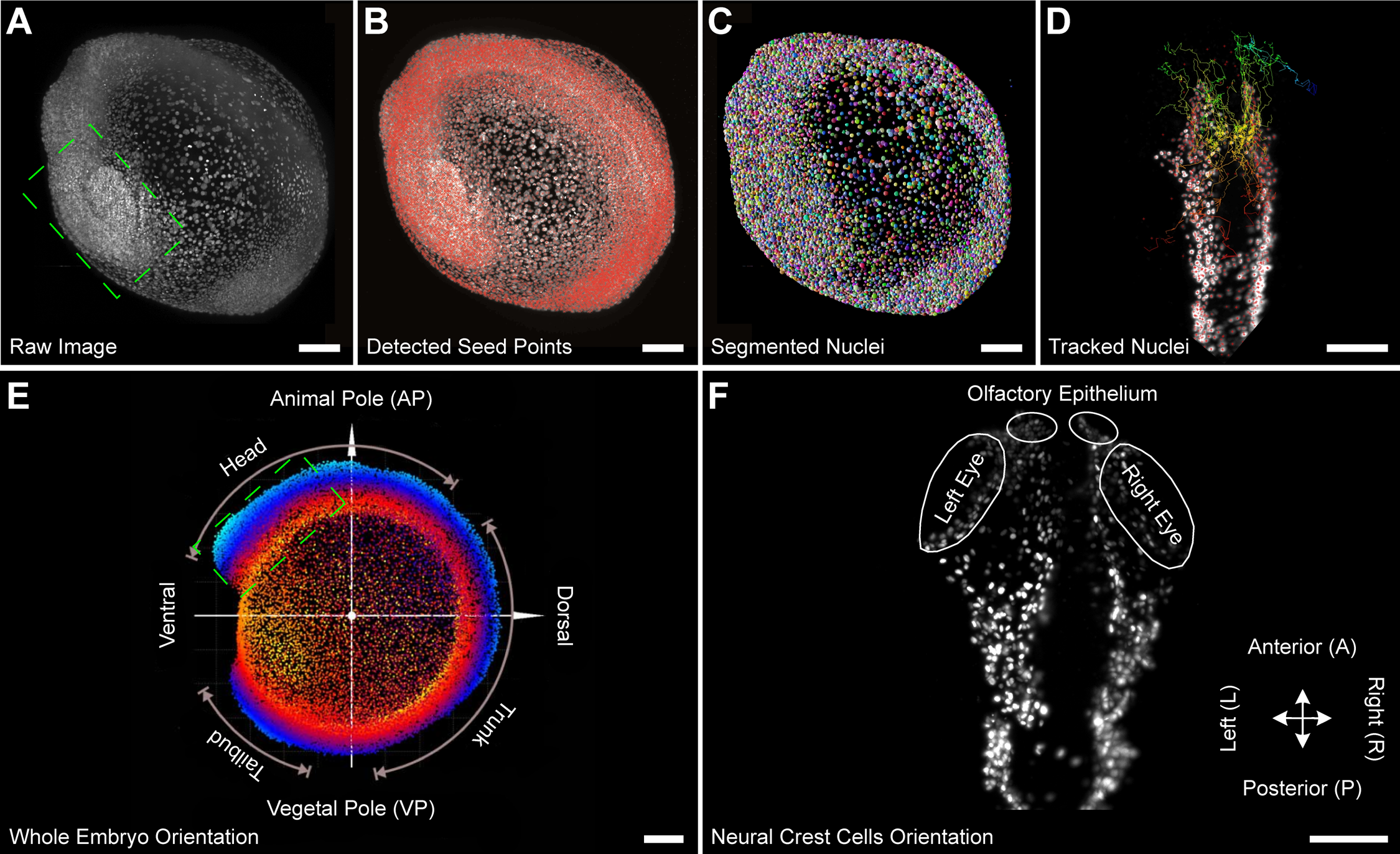

Supplement: S1 Fig — (A) Maximum intensity projection of a single time point of an entire zebrafish embryo at early somitogenesis stages [11, 66]. (B) Maximum intensity projection of a 3D light-sheet microscopy image with superimposed centroids of detected cell nuclei in red. (C) Volume rendering of segmented cell nuclei using a random color code. (D) Exemplary movement paths for 25 arbitrarily selected neural crest nuclei of a zebrafish embryo that were automatically tracked using a nearest neighbor algorithm [58, 59]. Dorsal view of the anterior half of the embryo (anterior up). All data sets were aligned prior to the analysis, to obtain a common reference orientation. (E) Whole-embryo data sets were oriented such that the animal-vegetal axis was aligned with the y-axis (animal pole on the positive y-axis) and the dorsoventral axis was aligned with the x-axis (dorsal part on the positive x-axis). (F) The embryos highlighting neural crest cells were oriented such that the anteroposterior axis formed a left-right symmetry axis with the head region on the positive y-axis (dorsal view, anterior up). Circled regions in (F) indicate the positions of the prospective eyes and the olfactory epithelium of the embryo, respectively. The green rectangle in panels (A) and (E) indicates the neural crest cell region visualized in (F). Panels (A)-(D) were adapted from [67] and panel (E) was adapted from [11]. Scale bar: 100 μm. (TIF) [file pcbi.1006128.s004.tif]

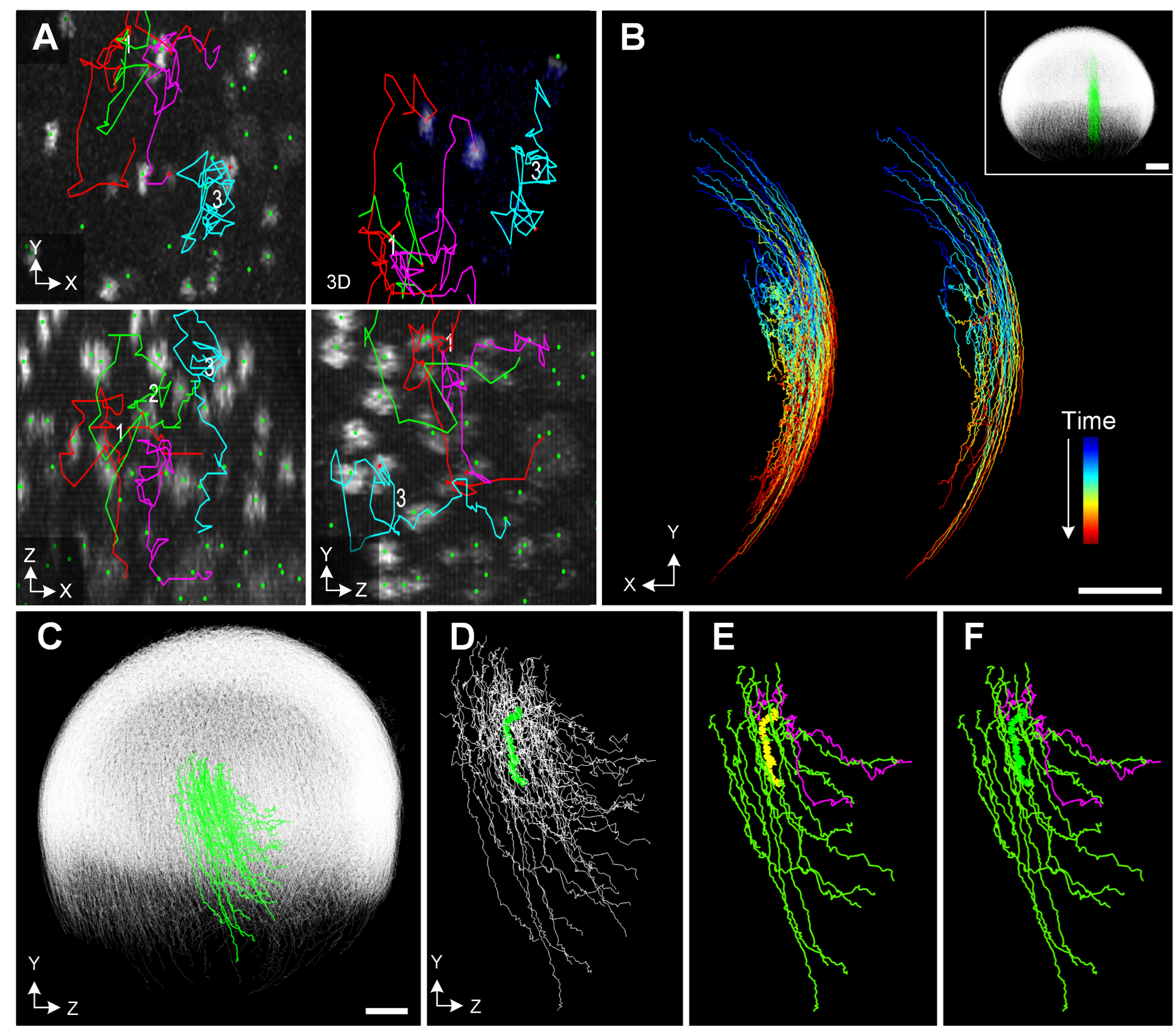

Supplement: S2 Fig — (A) Erroneous tracks can be interactively repaired to obtain a set of corrected full-length tracks. The curation mode features interactive maximum intensity projections and 3D volume rendering with superimposed cell tracks. Moreover, link candidate predictions and different curation modes like breadth-first or depth-first curation strategies minimize the required manual correction effort. (B-F) As an alternative to manually correcting the entire tracking database, we propose to select only a subset of sufficiently long tracks (B, e.g., select only tracks spanning over 70% of the time interval of interest), to perform the region of interest selection on these corrected tracks (C-E) and to finally assign all unlabeled tracks to the predominating group of their spatiotemporally nearest neighbors (F). Panel (A) shows a C. elegans embryo of the Cell Tracking Challenge that was imported to EmbryoMiner (data set by the Waterston lab, The George Washington University, Washington D.C., USA) [24, 36] and panels (B-F) show cropped regions of a whole-embryo zebrafish data set (S2 Note). (TIF) [file pcbi.1006128.s005.tif]

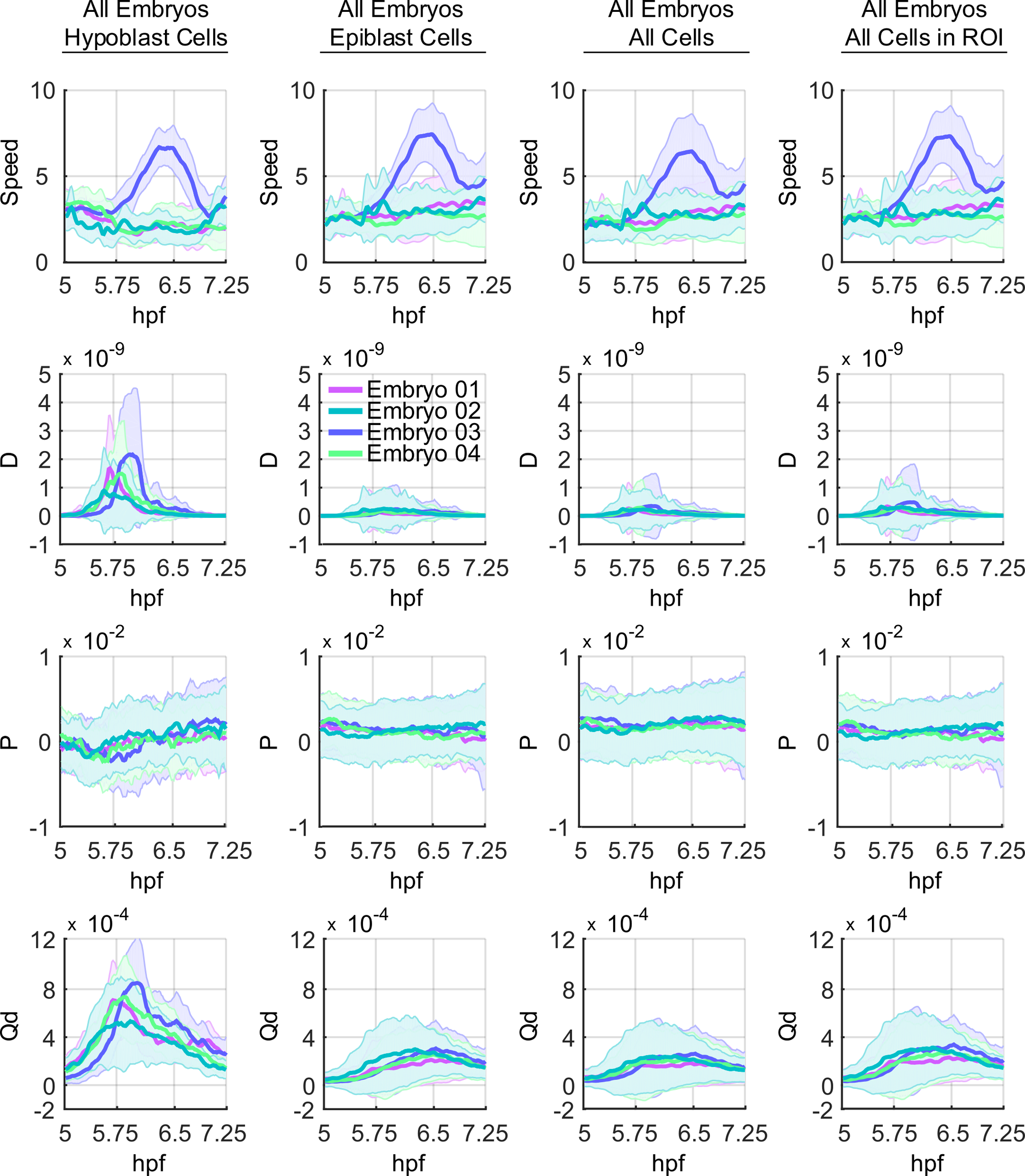

Supplement: S3 Fig — The extracted features are identical to the ones depicted in Fig 5 but all embryos are shown in a single plot for easier comparison. Except for the speed feature of Embryo 03 that was affected by the movement during image acquisition, all other quantitative features were nicely captured and revealed comparable patterns among all analyzed embryos and groups. The group selections in the last two columns show that properly selected regions are crucial to avoid the superposition of different movement behaviors. (TIF) [file pcbi.1006128.s006.tif]

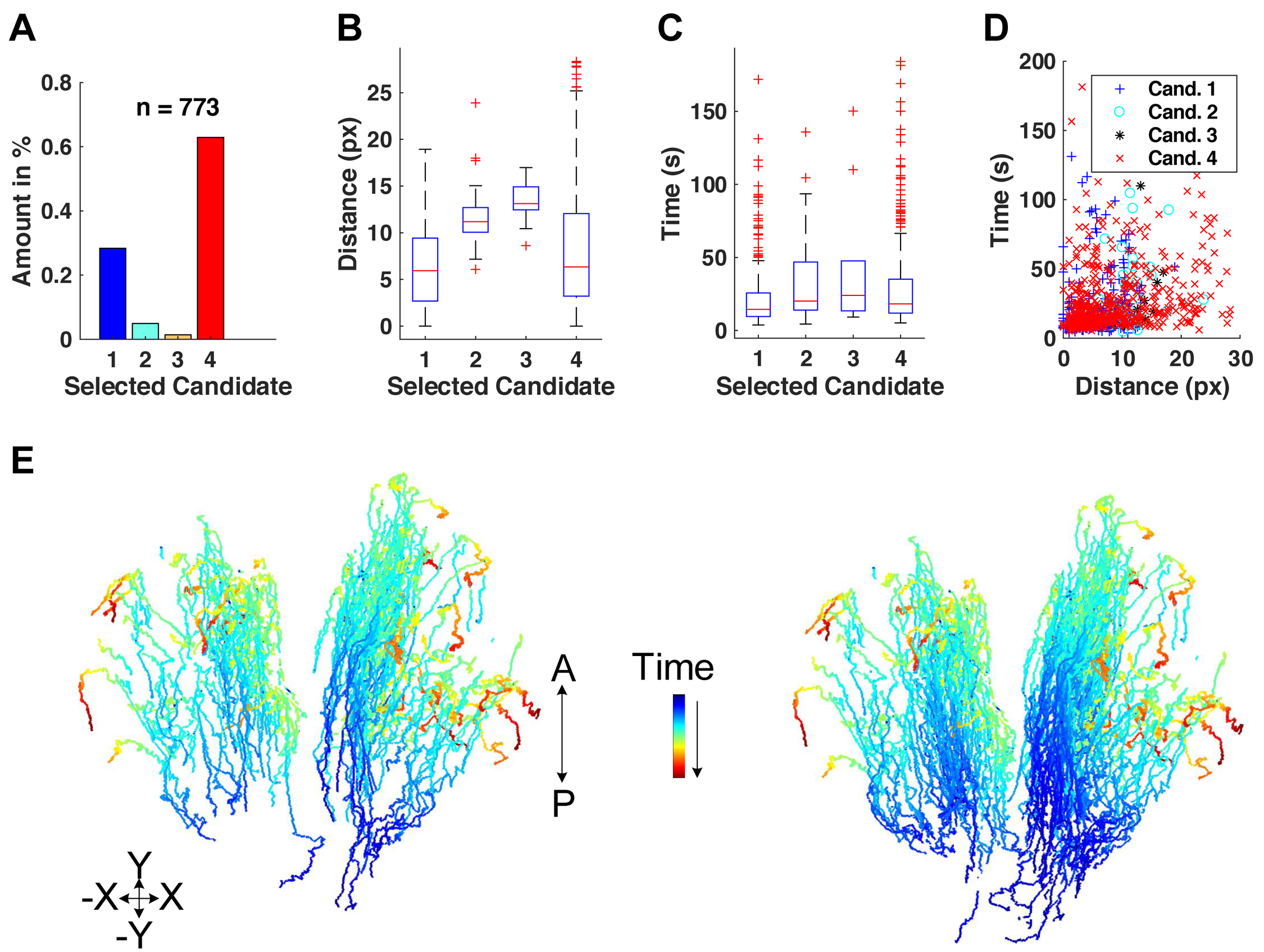

Supplement: S4 Fig — (A) Distribution of the selected correct link candidate, where Candidates 1-3 are the automatic suggestions computed by the framework and Candidate 4 indicates that the user selected an alternative link candidate to be correct. (B) Box plots of the distance in pixel of the selected link candidate. (C) Box plot of the time in seconds that was required by the manual annotator to perform the decision. For box plots in (B) and (C), the median is indicated by red bar and the 25% and 75% quantiles are indicated by the lower and upper extents of the box, respectively. (D) Scatter plot of time in seconds versus the distance of the linked candidate. Color and symbols indicate, which candidate has been selected. (E) Selected trajectories of the neural crest data set before (left) and after the manual curation (right). Time is indicated by the color-code from blue to red. (TIF) [file pcbi.1006128.s007.tif]
